# The incidence of endophthalmitis or macular involvement and the necessity of a routine ophthalmic examination in patients with candidemia

**DOI:** 10.1371/journal.pone.0216956

**Published:** 2019-05-23

**Authors:** Takashi Ueda, Yoshio Takesue, Issei Tokimatsu, Taiga Miyazaki, Nana Nakada-Motokawa, Miki Nagao, Kazuhiko Nakajima, Hiroshige Mikamo, Yuka Yamagishi, Kei Kasahara, Shingo Yoshihara, Akira Ukimura, Koichiro Yoshida, Naomi Yoshinaga, Masaaki Izumi, Hiroshi Kakeya, Koichi Yamada, Hideki Kawamura, Kazuo Endou, Kazuaki Yamanaka, Mutsunobu Yoshioka, Kayoko Amino, Hiroki Ikeuchi, Motoi Uchino, Yoshitsugu Miyazaki

**Affiliations:** 1 Department of Infection Control and Prevention, Hyogo College of Medicine, Nishinomiya, Japan; 2 Division of Clinical Infectious Diseases, Department of Medicine, School of Medicine, Showa University, Tokyo, Japan; 3 Department of Infection Prevention and Control, Kobe University Hospital, Kobe, Japan; 4 Department of Respiratory Medicine, Nagasaki University Hospital, Nagasaki, Japan; 5 Department of Infection Control and Prevention, Kyoto University Hospital, Kyoto, Japan; 6 Department of Clinical Infectious Diseases, Aichi Medical University, Aichi, Japan; 7 Center for Infectious Diseases, Nara Medical University, Nara, Japan; 8 Infection Control Center, Osaka Medical College Hospital, Osaka, Japan; 9 Kindai University Hospital, Osaka, Japan; 10 Department of Internal Medicine, Kansai Rosai Hospital, Amagasaki, Japan; 11 Department of Infection Control Science, Graduate School of Medicine, Osaka City University, Osaka, Japan; 12 Department of Infection Control and Prevention, Division of Medical and Environmental Safety, Kagoshima University Hospital, Kagoshima, Japan; 13 Hyogo Prefectural Amagasaki General Medical Center, Amagasaki, Japan; 14 Department of Urology, Hyogo Prefectural Nishinomiya Hospital, Nishinomiya, Japan; 15 Department of Pharmacy, Takarazuka City Hospital, Takarazuka, Japan; 16 Dentistry and Oral and Maxillofacial Surgery, Nishinomiya Central Municipal Hospital, Nishinomiya, Japan; 17 Department of Inflammatory Bowel Disease, Hyogo College of Medicine, Nishinomiya, Japan; 18 Department of Chemotherapy and Mycoses, National Institute of Infectious Diseases, Shinjuku-ku, Tokyo, Japan; Louisiana State University, UNITED STATES

## Abstract

**Background:**

The incidence of ocular candidiasis (OC) in patients with candidemia varies across different reports, and the issue of whether routine ophthalmoscopy improves outcomes has been raised. This study investigated the incidence of OC and evaluate whether the extent of OC impacts the clinical outcomes.

**Methods:**

This retrospective study included non-neutropenic patients with candidemia who underwent treatment at one of 15 medical centers between 2010 and 2016. Chorioretinitis without other possible causes for the ocular lesions and endophthalmitis was classified as a probable OC. If signs of chorioretinitis were observed in patients with a systemic disease that causes similar ocular lesions, they were classified as a possible OC.

**Results:**

In total, 781 of 1089 patients with candidemia underwent an ophthalmic examination. The prevalence of OC was 19.5%. The time from the collection of a positive blood culture to the initial ophthalmic examination was 5.0 ± 3.9 days in patients with OC. The leading isolate was *Candida albicans* (77.9%). Possible OC was associated with unsuccessful treatments (resolution of ocular findings) (odds ratio: 0.354, 95% confidence interval: 0.141–0.887), indicating an overdiagnosis in patients with a possible OC. If these patients were excluded, the incidence fell to 12.8%. Endophthalmitis and/or macular involvement, both of which require aggressive therapy, were detected in 43.1% of patients; a significantly higher incidence of visual symptoms was observed in these patients.

**Conclusion:**

Even when early routine ophthalmic examinations were performed, a high incidence of advanced ocular lesions was observed. These results suggest that routine ophthalmic examinations are still warranted in patients with candidemia.

## Introduction

*Candida* species are the fourth most common nosocomial bloodstream organism [1], and ocular involvement is reported as one of the main complications in patients with candidemia [2,3]. Oude Lashof et al. [4] reported that 16% of patients with candidemia had ocular candidiasis (OC). Nagao et al. [5] reported that 26.5% of patients with candidemia had findings consistent with those of OC. Krishna et al. [6] reported that the overall incidence of OC was 26% in their study. Based on the considerably high incidence of OC in patients with candidemia, current guidelines [2, 3] recommended an ophthalmological examination for all patients with candidemia. In contrast, lower rates of OC ranging from 2.9% to 9.7% were recently reported [7–11], and the necessity of a routine ophthalmology consultation to rule out ocular involvement in patients with candidemia has consequently been challenged.

There are two types of OC: chorioretinitis, which is associated with a lesion restricted to the choroid and retina, and endophthalmitis, which is associated with a lesion extending into the vitreous body [2]. An in-depth search for sight-threatening lesions near the macula or that invade the vitreous body should be performed to select the appropriate treatment option (e.g. choice of antifungals, intravitreal injection, and/or vitrectomy) [2]. We previously developed management bundles in non-neutropenic patients with candidemia [12] that included a routine ophthalmological examination to rule out OC. Using the bundles as a check-list, we performed ophthalmological examination in patients with candidemia. The aim of this study was to estimate the incidences of OC in patients with candidemia, and to estimate the incidence of endophthalmitis (or macular involvement) in patients with OC who underwent a comprehensive examination. We also investigated how the extent of ocular infection impacted the clinical outcomes of patients with OC.

## Materials and methods

### Ethics statement

This study was approved by the institutional review boards of Hyogo College of Medicine (No. 2599) and of each participating facility [Kobe University Graduate School of Health Sciences (No. 472-3); Nagasaki University Hospital (17061914); Kyoto University Hospital (R2300); Aichi Medical University Hospital (2017-H072); Nara Medical University (No.1624), Osaka Medical College (No.2199); Kindai University Faculty of Medicine (No. 29-029); Graduate School of Medicine, Osaka City University (No. 3813); Kagoshima University (No. 170113); Hyogo Prefectural Amagasaki General Medical Center (No.29-8); Hyogo Prefectural Nishinomiya Hospital (H29-3); and Takarazuka City Hospital (No. 201631)]. The institutional review board waived the requirement for informed consent from patients included in this study. Ethics approval was the responsibility of each participating center. If necessary, investigators obtained formal approval of the protocol by the regional ethics committee.

### Surveillance population and diagnosis of ocular candidiasis

This retrospective study included non-neutropenic patients (>17 years of age) with candidemia who underwent treatment at one of 15 medical centers in Japan between 2010 and 2016. Included patients underwent at least one dilated fundoscopic examination performed by an ophthalmologist. The diagnosis of OC was made based on the definition previously formulated by Oude Lashof et al. [4] *Proven* OC was defined as ocular lesions that occur in combination with a positive histology or culture of a vitreous aspirate. Either endophthalmitis, which is associated with a lesion extending into the vitreous body, or chorioretinitis without other possible causes for the ocular lesions, was classified as a probable case of OC. Although a diagnosis of OC was made by an ophthalmologist, signs of chorioretinitis in patients with an underlying systemic disease that causes similar lesions such as diabetes, hypertension, or concomitant bacteremia, led retrospectively to a diagnosis of possible OC.

### Collection of ophthalmologic data

The following parameters were reviewed: whether an ophthalmological examination was performed or not in patients with candidemia, the incidence of OC, the timing of the diagnosis, the subsequent development of OC in patients without OC upon initial examination, lesion extension into the vitreous body and macular involvement, initial antifungal treatments, and two measures of clinical outcomes (successful treatment and 28-day mortality). Antifungal therapy was demonstrated as the first systemic antifungal treatments administered after diagnosis of candidemia and OC. The treatment of OC was considered successful when the resolution of the lesions was observed during a follow-up ophthalmological examination. Patients who had at least 2 weeks of follow-up after OC diagnosis were included for the evaluation of clinical outcome. Variables associated with successful treatment were also identified in univariate and multivariate analyses.

### Statistical methods

The relative risk ratio and 95% confidence interval (CI) were estimated for each variable using the chi-squared test and potential confounders were examined using cross-tabulation. Variables identified as potentially relevant by these univariate analyses (p < 0.1) were subsequently entered into a logistic regression model to estimate the size of the association [odds ratio (OR)] and the 95% confidence interval (CI). SPSS ver. 24.0 for Windows (Chicago, IL) was used for all analyses and the level of significance was set at p < 0.05.

## Results

### Incidence of ocular candidiasis

In total, 781 of 1089 patients (71.7%) with candidemia underwent an ophthalmologic examination to rule out ocular involvement. The prevalence of OC in our sample was 19.5%. Vitreous body sampling was not performed in any patient. Although proven OC was not diagnosed in any patient, 100 patients were classified as having probable OC, 51 were classified as having possible OC. One patient’s medical record did not state whether the lesion extended into the vitreous body (indeterminant case). Upon initial ophthalmologic examination, 133 patients had OC (88 probable, 44 possible, and one indeterminant). Follow-up examinations were performed on 279 (43.1%) of the remaining 648 patients; of these, 19 had OC (12 probable, seven possible). The time from the collection of a positive blood culture to the initial ophthalmic examination was 5.0 ± 3.9 days in patients with OC. In the 133 patients diagnosed with OC during the initial examination, the mean time from positive blood culture to OC diagnosis was 5.1 ± 4.0 days. In the 19 patients diagnosed with OC only on follow up eye examination, the mean time from positive blood culture to the initial examination was 3.8 ± 2.8 days, and the mean time from positive blood culture to OC diagnosis was 12.6 ± 5.1 days.

### Isolated *Candida* species

A total of 154 strains were isolated from the blood cultures. The most commonly found *Candida* species was *C*. *albicans* (77.9%), followed by *C*. *glabrata* and *C*. *parapsilosis* (8.4% in each), *C*. *tropicalis* (3.9%), *C*. *krusei* (0.6%), and other *Candida* spp. (0.6%).

### Endophthalmitis and macular involvement

With regards to the extent of the ocular infection, 151 patients except for one indeterminate case were analyzed. Thirty-two patients (21.2%) had endophthalmitis, whereas macular involvement was involved in 47 patients, of which 14 patients had concomitant vitritis (Table 1). Baseline characteristics of the patients with OC are presented as a function of the extent of ocular lesions in Table 2. There was no significant difference in the rate of delayed diagnosis (≥ 2 weeks after the blood culture collection) between patients with endophthalmitis and those with chorioretinitis (9.4% vs. 8.4%, p = 0.856). The mean time from a positive blood culture collection to the diagnosis of the ocular disease was 6.7 ± 5.9 days in patients with endophthalmitis and 5.9 ± 4.5 days in patients with chorioretinitis. Six of the 32 patients with endophthalmitis did not receive a diagnosis during the initial examination. Of the 19 patients diagnosed during follow-up examination, six (31.6%) had endophthalmitis, and one (5.3%) had macula-threatening chorioretinitis. The mean lengths of follow-up examinations in these patients, from the points of positive blood cultures and initial ophthalmologic examinations, were 13.8 ± 7.2 days and 9.3 ± 3.7 days, respectively in patients with endophthalmitis, and they were 16 days and 14 days, respectively, in the patient with macula-threatening chorioretinitis. Visual symptoms were reported in 37 of 120 conscious patients (30.8%). The rate of visual symptoms was significantly higher in patients who had chorioretinitis with macular involvement (46.2%) and those with endophthalmitis (67.7%) compared with patients who had chorioretinitis, without macular involvement (4.8%) (both p’s < 0.001). Visual symptoms at last ophthalmologic follow-up were reported in four of 25 patients (16.0%) with endophthalmitis (duration of follow-up: 28 to 90 days after OC diagnosis).

**Table 1:** Lesion extensions into vitreous body and macular involvement in patients with ocular candidiasis.

	No. of patients (prevalence in patients with ocular candidiasis)		
	without macular involvement	with macular involvement	Total
Chorioretinitis	86 (57.0%)	33 (21.9%)	119 (78.8%)
Endophthalmitis	18 (11.9%)	14 (9.3%)	32 (21.2%)
Total	104 (68.9%)	47 (31.1%)	151 (100%)

**Table 2:** Baseline characteristics of patients with ocular candidiasis as a function of the extent of ocular disease.

	Chorioretinitis without macular involvement (n = 86)	Chorioretinitis with macular involvement (n = 33)	P-value^¶^	Endophthalmitis (n = 32)	P-value^¶^
Sex (male)	55 (64.0%)	19 (57.6%)	0.521	13 (40.6%)	0.023
Age (>65 years)	54 (62.8%)	19 (57.6%)	0.55	23 (71.9%)	0.357
Body mass index (<18.5)	30 (34.9%)	12 (36.4%)	0.88	10 (31.3%)	0.711
Total parenteral nutrition	58 (67.4%)	19 (57.6%)	0.313	23 (71.9%)	0.645
Steroid use	23 (26.7%)	9 (27.3%)	0.954	10 (31.3%)	0.628
Immunosuppressive therapy	7 (8.1%)	5 (15.2%)	0.255	4 (12.5%)	0.487
Anticancer therapy	8 (9.3%)	2 (6.1%)	0.724	3 (9.4%)	1.000
Surgery (within 28 days)	32 (37.2%)	9 (27.3%)	0.307	10 (31.3%)	0.548
Digestive tract	22 (25.6%)	5 (15.2%)	0.224	8 (25.0%)	0.949
Others	10 (11.6%)	4 (12.1%)	0.94	2 (6.3%)	0.39
Malignant tumor	36 (41.9%)	12 (36.4%)	0.584	17 (53.1%)	0.274
Solid cancer	35 (40.7%)	10 (30.3%)	0.295	17 (53.1%)	0.227
Hematological malignancy	1 (1.2%)	2 (6.1%)	0.185	0 (0.0%)	1.000
Serum Albumin <2.8 g/dL	60 (69.8%)	24 (72.7%)	0.751	18 (56.3%)	0.168
Diabetes	14 (16.3%)	9 (27.3%)	0.173	3 (9.4%)	0.556
Hypertension/heart disease	27 (31.4%)	7 (21.2%)	0.271	4 (12.5%)	0.058
Chronic hepatic dysfunction	11 (12.8%)	4 (12.1%)	1.000	5 (15.6%)	0.689
Chronic renal failure	19 (22.1%)	8 (24.2%)	0.802	6 (18.8%)	0.693
Organ transplantation	0 (0.0%)	0 (0.0%)	-	0 (0.0%)	-
Inflammatory bowel disease	7 (8.1%)	3 (9.1%)	1.000	3 (9.4%)	0.83
Prolonged ICU stay	18 (20.9%)	9 (27.3%)	0.46	5 (15.6%)	0.518
Ventilator use	27 (31.4%)	9 (27.3%)	0.661	4 (12.5%)	0.058
APACHE II score ≥ 15	36 (41.9%)	13 (39.4%)	0.807	6 (18.8%)	0.02
Diagnosis in initial examination	74 (86.0%)	32 (97.0%)	0.109	26 (81.3%)	0.568
Delayed diagnosis (≧2 weeks of blood culture)	6 (7.0%)	4 (12.1%)	0.461	3 (9.4%)	0.702
Isolated *Candida species*					
*C*. *albicans*	65 (75.6%)	27 (81.8%)	0.467	27 (84.4%)	0.306
*C*. *glabrata*	10 (11.6%)	2 (6.1%)	0.507	1 (3.1%)	0.285
*C*. *parapsilosis*	7 (8.1%)	5 (15.2%)	0.31	1 (3.1%)	0.445
Other *Candida* spp	5 (4.7%)	0 (0.0%)	0.366	3(6.3%)	0.785

^¶^: vs. Chorioretinitis without macula involvement

ICU: intensive care unit; APACHE II: acute physiology and chronic health evaluation II

### Selected antifungals and duration of therapy

The first systemic antifungal treatments administered after the diagnosis of candidemia were echinocandins in 120 patients (78.9%), fluconazole/voriconazole in 26 patients (17.1%), and liposomal amphotericin B (L-AMB) in six patients (3.9%). In contrast, the selected antifungals for the initial treatment of OC were fluconazole/voriconazole in 79 patients (52.0%), liposomal amphotericin B (L-AMB) in 45 patients (29.6%), and echinocandins in 29 patients (19.1%). Echinocandins were initially used in 120 patients after the diagnosis of candidemia; this treatment was changed to fluconazole/voriconazole in 59 patients and L-AMB in 33 patients, respectively, after the diagnosis of OC. In one patient who was treated initially by L-AMB, echinocandins were used after the diagnosis of OC because of the deterioration of renal function. Combination therapy consisting flucytosine and L-AMB was administered in 15.1% of patients. Step-down oral therapy using azole was administered in 59 of 152 patients (38.8%) (intravenous antifungals before the administration of an oral formulation of azole: azole, 34 patients; L-AMB, 18 patients; and echinocandins, seven patients). Azoles were administered orally after intravenous initial loading dose in none of the patients.

Two patients received intravitreal injections of antifungal drugs. Vitrectomy was not performed in any patient. The rate of azole use was significantly lower and the rate of L-AMB use was significantly higher in patients with endophthalmitis or macular involvement than in those who had chorioretinitis without macular involvement (Table 3). Among the 32 patients with endophthalmitis, 16 patients (50.0%) received L-AMB and 11 (34.4%) received combination therapy of L-AMB and flucytosine during the overall treatment. When evaluating the effect of therapy duration, patients who had no ophthalmology appointments after discharge (n = 16) and patients who passed away before undergoing 4 weeks of therapy (n = 32) were excluded from the analysis. The total duration of therapy was 48.7 ± 30.5 days; therapy ≥ 4 weeks in length was administered in 81 of 104 patients (77.9%). The total duration of therapy was significantly longer in patients who had endophthalmitis than in patients who had chorioretinitis without macular involvement (62.6 ± 37.7 vs. 41.5 ± 22.8 days, p = 0.003) (Table 4).

**Table 3:** Initially selected antifungal agents as a function of the extent of ocular infection.

Antifungal agents	All (n = 152^¶^)	Chorioretinitis without macular involvement (n = 86)	Chorioretinitis with macular involvement (n = 33)	Endophthalmitis (n = 32)	Endophthalmitis or macular involvement (n = 65)	P-value*
Fluconazole/voriconazole	79 (49.3%)	51 (59.3%)	13 (39.4%)	14 (43.8%)	27 (41.5%)	0.03
Echinocandin	29^&^ (19.1%)	16^&^ (18.6%)	6 (18.2%)	7 (21.9%)	13 (20.0%)	0.83
Liposomal amphotericin B	45 (29.6%)	20 (23.3%)	14 (42.4%)	11 (34.4%)	25 (38.5%)	0.04
Flucytosine combined with liposomal amphotericin B	23 (15.1%)	9 (10.5%)	6 (18.2%)	8 (25.0%)	14 (21.5%)	0.06

^¶^: For one patient, it was not determined whether the lesion had extended into the vitreous body

^&^: Combination therapy with fluconazole was administered to one patient

*: Chorioretinitis without macular involvement vs. endophthalmitis or macular involvement

**Table 4:** Duration of therapy in patients with ocular candidiasis as a function of the extent of ocular infection.

Antifungal therapy	Duration of treatment (days)				
	Chorioretinitis		P-value^¶^	Endophthalmitis (n = 25)	P-value^¶^
	without macular involvement (n = 55)	with macular involvement (n = 24)			
Intravenous treatment (days)^&^	30.8±19.9	32.4±13.0	0.202	49.5±28.1	0.001
Entire treatment course including oral antifungals (days)	41.5±22.8	50.6±33.4	0.166	62.6±37.7	0.003

^¶^: vs. chorioretinitis without macular involvement

^&^: All patients treated initially with intravenous antifungals

### Clinical outcomes

Clinical outcomes in patients with OC are shown as a function of the extent of OC in Table 5. Sixteen patients were excluded from the analysis of successful treatment (resolution of ocular findings) because a follow-up ophthalmology examination was not performed at least 2 weeks of antifungal treatment specifically for the OC. The average follow-up days for eye findings after diagnosis of OC in patients with endophthalmitis was significantly longer than that in patients with chorioretinitis without macular involvement. Successful treatment of OC was achieved in 110 of 136 patients (80.1%). The 28-day mortality rate was 21.1%. There was no significant difference in the successful treatment rate and mortality rate between patients who had chorioretinitis without macula involvement and patients who had chorioretinitis with macular involvement or those who had endophthalmitis. There is a significant difference in the successful treatment rate between possible chorioretinitis and probable chorioretinitis [68.2% (30/44 patients) vs. 88.7% (55/62 patients), p = 0.009].

**Table 5:** Clinical outcomes and follow-up days in patients with ocular candidiasis as a function of the extent of ocular infection.

Clinical Outcomes	No of patients with Chorioretinitis		P-value^¶^	No of patients with endophthalmitis	P-value^¶^
	without macular involvement	with macular involvement			
Successful treatment	58/75 (77.3%)	27/31(87.1%)	0.296	24/29 (82.8%)	0.605
28-day mortality	22/86 (25.6%)	6/33 (18.2%)	0.394	4/32 (12.5%)	0.127
The average number of follow-up days after diagnosis of ocular candidiasis (days)	38.7 ± 30.6	48.5 ± 38.9	0.224	52.1 ± 37.4	0.044

^¶^: vs. chorioretinitis without macular involvement

### Factors associated with successful treatment

The univariate analyses identified several factors that decreased the rate of successful treatment, which included possible OC, chronic renal failure, prolonged intensive care unit stay, ventilator use, and an acute physiology and chronic health evaluation II (APACHE II) score of ≥ 15 (Table 6). Because of potential confounds, ventilator use was excluded from the multivariate analysis. The resulting independent factors associated with unsuccessful treatment were possible ocular candidiasis (adjusted OR: 0.354, 95% CI: 0.141–0.887) and chronic renal failure (adjusted OR: 0.216, 95% CI: 0.081–0.580).

**Table 6:** Factors associated with successful treatment in patients with ocular candidiasis.

Factors	No of patients with successful treatment (%)		Crude odds ratio (95%CI) of significant factors	P-value	Adjusted odds ratio (95%CI) of significant factors	P-value
	Patients with factor	Patients without factor				
Possible ocular candidiasis	30/44 (68.2%)	79/91 (86.8%)	0.325 (0.135–0.783)	0.010	0.354 (0.141–0.887)	0.027
Chorioretinitis without macula involvement	58/75 (77.3%)	51/60 (85.0%)				
Chorioretinitis with macula involvement	27/31 (87.1%)	82/104 (78.8%)				
Endophthalmitis	24/29 (82.8%)	85/106 (80.2%)				
Azole	55/69 (79.7%)	54/66 (81.8%)				
Echinocandin	22/26 (84.6%)	87/109 (79.8%)				
Liposomal amphotericin B	32/41 (78.0%)	77/94 (81.9%)				
Combination therapy with flucytosine	18/20 (90.0%)	91/115 (79.1%)				
Diagnosis in initial examination	98/119 (82.4%)	11/16 (68.8%)				
Delayed diagnosis (≧2 weeks of blood culture)	10/13 (76.9%)	99/122 (81.1%)				
Non-albicans	23/30 (76.7%)	86/105 (81.9%)				
Sex (male)	59/78 (75.6%)	50/57 (87.7%)				
Age (>65 years)	68/81 (84.0%)	41/54 (75.9%)				
Body mass index (<18.5)	34/45 (75.6%)	75/90 (83.3%)				
Total parenteral nutrition	71/87 (81.6%)	38/48 (79.2%)				
Steroid use	30/38 (78.9%)	79/97 (81.4%)				
Immunosuppressive therapy	12/13 (92.3%)	97/122 (79.5%)				
Anticancer therapy	8/10 (80.0%)	101/125 (80.8%)				
Surgery (within 28 days)	35/44 (79.5%)	74/91 (81.3%)				
Malignant tumor	46/57 (80.7%)	63/78 (80.8%)				
Serum Albumin <2.8 g/dL	74/89 (83.1%)	35/46 (76.1%)				
Diabetes	15/21 (71.4%)	94/114 (82.5%)				
Hypertension/heart disease	22/30 (73.3%)	87/105 (82.9%)				
Chronic hepatic dysfunction	11/16 (68.8%)	98/119 (82.4%)				
Chronic renal failure	14/25 (56.0%)	95/110 (86.4%)	0.201 (0.129–0.524)	0.001	0.216 (0.081–0.580)	0.002
Organ transplantation	0/0 (0.0%)	109/135 (80.7%)				
Inflammatory bowel disease	12/13 (92.3%)	97/122 (79.5%)				
Prolonged ICU stay	18/28 (64.3%)	91/107 (85.0%)	0.316 (0.133–0.809)	0.013		
Ventilator use	19/32 (59.4%)	90/103 (87.4%)	0.211 (0.134–0.527)	<0.001		
APACHE II score ≥15	30/45 (66.7%)	79/90 (87.8%)	0.278 (0.135–0.674)	0.003		

95% CI: 95% confidence interval; APACHE II: Acute physiology and chronic health evaluation II

## Discussion

Overall, the incidence of OC in patients with candidemia was 19.5%, which is consistent with that of prior studies in Japan [5, 13]. Nineteen of 152 patients with OC were diagnosed at the time of follow-up examination, which illustrates the importance of follow-up ophthalmologic examinations in patients with candidemia. In patients diagnosed with OC only on follow up eye examination, early initial examination after positive blood cultures (i.e., 3.8 days) might yield a negative diagnosis with respect to OC. Of the patients with OC, 77.9% were infected with *C*. *albicans*. This result is consistent with prior reports. For instance, Oude Lashof et al. [4] found that patients with OC were significantly more often infected with *C*. *albicans* and less often infected with *C*. *parapsilosis* than patients without OC. Lingappan et al. [14] demonstrated that the most prevalent organism was *C*. *albicans* (33 of 38 patients with ocular candidiasis). Blennow et al. [7] described that *C*. *albicans* was isolated from a blood culture in 11 of 12 patients with OC, compared with 27 of 48 patients without OC; they furthermore suggested that *C*. *albicans* was an independent risk factor for OC. Finally, Nagao et al. [5] reported that *C*. *albicans* bloodstream infections and higher β-D-glucan values were independent risk factors for OC.

Independent factors associated with unsuccessful treatment in the present study were possible OC and chronic renal failure. Successful treatment was defined as the resolution of the ocular lesions. If signs of chorioretinitis were observed in patients with an underlying systemic disease, these cases were classified as possible OC, regardless of the patient’s diagnosis by an ophthalmologist. Underlying systemic diseases may cause clinical failure. However, ocular lesions caused by systemic disease can be included in possible OC. Retinal lesions caused by systemic diseases cannot be resolved with antifungal agents, and there is a risk of overdiagnosis among patients with possible OC. Probable chorioretinitis was diagnosed in patients with deep focal white infiltrates in the retina. In addition, cases with hemorrhages, Roth spots, or cotton wool spots, were classified as probable in patients who had no other reason for retinal lesions based on the definition formulated by Oude Lashof et al. [4]

In contrast, Donahue et al. [10] made a clear differentiation between candida chorioretinitis characterized by deep white infiltrative chorioretinal lesions and chorioretinitis characterized by nonspecific lesions that include hemorrhages, Roth spots, or cotton wool spots. These nonspecific lesions may have different etiologies that include vascular nonperfusion and hypertension. Current guidelines [2, 3] recommend that treatment should be continued until the complete resolution of ocular lesions. However, given the risk of over-diagnosing OC, this rule might not apply to all patients with possible OC. A two-to-three week treatment course following the clearance of candidemia is considered to be sufficient in patients with an underlying systemic disease that causes nonspecific chorioretinal lesions (possible OC), especially if the systemic clinical signs caused by candidemia are resolved.

If patients with a possible OC diagnosis were excluded from our study, the incidence of OC would decrease from 19.5% to 12.8%, suggesting that their inclusion could potentially erroneously inflate the incidence rate. Using the same definition of OC, Oude Lashof et al. [4] reported that 16% of patients with candidemia had received a diagnosis of OC and 10.8% of patients had ophthalmological abnormalities that were consistent with the definition of probable OC. Similarly, Donahue et al. [10] reported that the incidence of ocular candidiasis was 9.3% and that an additional 20% of patients had nonspecific chorioretinal lesions not directly related to the candida infection.

Cure rates with antifungals were considered to be much lower in patients with endophthalmitis than in those with chorioretinitis [15]. With early recognition of OC, a high rate of L-AMB and combination therapy use, and a substantial duration of therapy, endophthalmitis was not found to be a risk factor for unsuccessful treatment in our study. However, as resolution of eye findings may take substantial time, significantly longer average follow-up for eye findings in patients with endophthalmitis would have caused better outcomes. Because the reliance on visual symptoms alone provides poor sensitivity in the diagnosis of OC, current guidelines [2, 3] indicate the importance of an ophthalmological examination prior to becoming clinically symptomatic to prevent the loss of visual acuity. However, the issue of whether the risk of missing OC outweighs the cost of ophthalmological examinations is still debated.

Several authors have stated that ocular involvement is uncommon and the clinical outcomes are not improved with an early routine ophthalmological examination [7–11]. Blennow et al. [7], for instance, reported that OC was not detected in patients with candidemia who had not received an initial ocular examination but that were subsequently examined after receiving two weeks of antifungal therapy. Gluck et al. [8] reported that ocular candidiasis was diagnosed in only one patient (2.9%) who had a risk factor for OC. Finally, Vena et al. [9] described that ocular lesions related to candidemia were found in only 7.7% of patients with candidemia, and ophthalmological findings led to a change in antifungal therapy in only 5.9% of cases. This finding led the authors to ask whether systematic ophthalmoscopy examinations were necessary. In agreement with this position, Donahue et al. [10] stated that only patients with risk factors for ocular involvement warrant an ophthalmological examination.

Antifungal therapy performed prior to the diagnosis of OC in patients with candidemia might prevent the vitreal extension of lesions, and it has been suggested that the development of endophthalmitis is uncommon in patients with candidemia [10]. Rodriguez-Adrian et al. [15] reported that the incidence of OC was only 1% in patients with candidemia. Similarly, Donahue et al. [10] did not diagnose endophthalmitis in any of the 118 examined patients with candidemia, and Krishna et al. [6] also did not report any cases of endophthalmitis either. Oude Lashof et al. [4] reported that the incidence of endophthalmitis was 1.6% in patients with candidemia and 10% in patients with OC. Finally, Khalid et al. [16] reported that the incidence of endophthalmitis was 1.4% in patients with candidemia and 11.1% in patients with OC.

However, 4.1% of patients with candidemia, and 21.2% of patients with OC were found to have endophthalmitis in our study. Nagao et al. [5] reported a similar incidence of endophthalmitis (18.5%) in Japanese patients. The rate of visual symptoms in our study was high in patients who had chorioretinitis with macular involvement and in those with endophthalmitis. In addition to the high incidence of endophthalmitis, chorioretinitis with macular involvement was found in 31.1% of patients in our study. Because of the high incidence of these invasive ocular lesions, visual symptoms were reported in 30.8% of conscious patients with OC. This result is inconsistent with that of a report by Oude Lashof, et al., who demonstrated that only one of 34 patients reported low visual acuity at baseline [4].

The present study has some limitations that should be considered. First, the current study was retrospective in nature. However, many participating institutions nonetheless used the bundles that recommended ophthalmologic examinations as part of the assessment checklist for the management of patients with candidemia. Second, in patients who had medical conditions that can be associated with retinal lesions, the accuracy of diagnosis of OC by an ophthalmologist might differ according to the institution where treatment was sought. Third, less than half of patients who were not diagnosed with OC during the initial examination underwent a second follow-up ophthalmological examination; this may have introduced a bias when assessing the incidence of OC. Fourth, although intravitreal injections of antifungal drugs are recommended in patients with macular involvement and endophthalmitis [2], only two patients received intravitreal injections of antifungal drugs. Lastly, although echinocandin was not recommended for the treatment of OC in the bundles [12], 19.2% of patients were treated with echinocandin. *C*. *glabrata* was isolated from blood culture in five of 29 patients in whom echinocandin was used, and there was no apparent reason for echinocandin use in the remaining 24 patients.

## Conclusions

The incidence of OC in our study was found to be approximately 20% in patients with candidemia. However, this may present an overestimation of the true incidence because of the inclusion of patients with a diagnosis of possible OC. If patients with possible OC were excluded, the incidence decreased to 12.8%. To evaluate the necessity of routine ophthalmological examinations in patients with candidemia, both the incidence of OC and the risk for treatment failure should be considered, particularly for patients who are examined only after manifesting ocular symptoms. Even with an early ophthalmological examination, advanced ocular lesions such as endophthalmitis and macular involvement—both of which may require aggressive therapy including an intravitreal antifungal injection or vitrectomy—were detected in 43% of patients. Our findings demonstrated that 21% of patients with OC were unconscious at the time of OC diagnosis, and that only 30% of conscious patients had visual symptoms. In addition, visual abnormalities were absent in one-third of patients with endophthalmitis and in half of the patients who had chorioretinitis with macular involvement. Current guidelines [2, 3] recommend echinocandins as the initial therapy for patients with candidemia. However, penetration of echinocandins into the vitreous body is poor [17]. Therefore, treatment regimens could be altered in a considerable number of patients when the diagnosis of OC is made. These results lead us to conclude that routine ophthalmology examinations are still warranted in patients with candidemia.

## Supporting information

S1 File Supporting information file 20190504.xlsx. (XLSX)
